# Supplementary material for: A multivariate decomposition analysis of drivers of overweight and obesity among Ghanaian women
Source: Commun Med (Lond). 2026 Jan 15;6:122. doi: 10.1038/s43856-026-01391-2 (PMC12917117; doi:10.1038/s43856-026-01391-2)
Supplement: Supplementary file 2 — Description of Additional Supplementary files [file 43856_2026_1391_MOESM2_ESM.pdf]

Description of Additional Supplementary Files

Supplementary Data 1: Prevalence estimates reflect survey-weighted proportions. Unweighted frequencies are omitted because raw counts do not correspond to the weighted population and could lead to misinterpretation. N = number of observations; SD = standard deviation; BMI = body mass index.

Supplementary Data 2: Trends in overweight and obesity among study participants, estimated using survey-weighted data. N = number of observations. GDHS = Ghana Demographic and Health Survey.

Supplementary Data 3: P-values were calculated using two-sided t-tests for regression coefficients, with standard errors adjusted for the survey design. Significance levels: *p>0.05, **p>0.01, ***p>0.001. All models are survey-weighted and account for the complex sampling design of the GDHS. Reference categories are indicated by a value of 1. aOR = adjusted odds ratio; CI = confidence interval; GDHS = Ghana Demographic and Health Survey.

Supplementary Data 4: P-values were calculated using two-sided t-tests for regression coefficients, with standard errors adjusted for the survey design. Significance levels: *p>0.05, **p>0.01, ***p>0.001. All models are survey-weighted and account for the complex sampling design of the GDHS. Reference categories are indicated by a value of 1. aOR = adjusted odds ratio; CI = confidence interval; GDHS = Ghana Demographic and Health Survey.

Supplementary Data 5: P-values were calculated using two-sided t-tests for regression coefficients, with standard errors adjusted for the survey design. Significance levels: *p>0.05, **p>0.01, ***p>0.001. All models are survey-weighted and account for the complex sampling design of the GDHS. Reference categories are indicated by a value of 1. aOR = adjusted odds ratio; CI = confidence interval; GDHS = Ghana Demographic and Health Survey.
